# Are Viruses Inhibited by APOBEC3 Molecules from Their Host Species?

**DOI:** 10.1371/journal.ppat.1000347

**Published:** 2009-04-24

**Authors:** Susan R. Ross

**Affiliations:** Department of Microbiology and Abramson Family Cancer Center, University of Pennsylvania, Philadelphia, Pennsylvania, United States of America; The Scripps Research Institute, United States of America

Organisms adapt to infectious agents by developing protective responses, and conversely, infectious agents develop adaptive countermeasures to these responses. Host defenses against infectious agents include adaptive and innate immune responses (e.g., natural killer cells, Toll-like receptors, and interferons). Recently, additional host defense systems against viruses have been identified. These include the TRIM [1], RIGI/MDA5 [2], Bst2/tetherin [3],[4], and APOBEC3 (A3) [5] proteins. Many of these anti-viral defense mechanisms were identified through the discovery of viral gene products that counteract their action, and thus it has been proposed that only viruses resistant to host-encoded restriction factors persist. However, recent in vivo work in the murine system has indicated that endogenous A3 proteins play important roles in limiting pathogenicity by murine viruses even though they only partially restrict infection [6]–[8]. Here I argue that these recent studies emphasize the critical importance of studying natural pathogenic viruses and host restriction factors in vivo.

**Figure 1:**
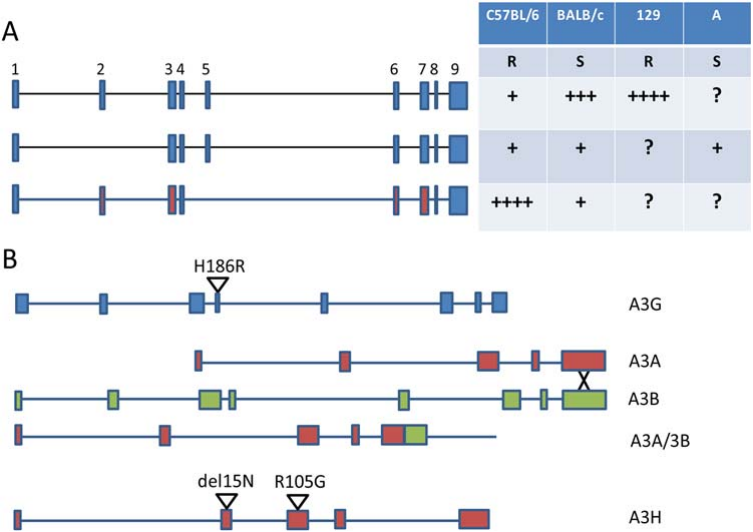
Polymorphisms in APOBEC 3 Genes. (A) Different splice variants and polymorphic exons found in different inbred mouse strains. Blue boxes denote exons found in inbred strains such as BALB/c and 129; red boxes denote exons with polymorphic amino acid differences in C57BL-derived strains. The table denotes the relative expression levels of the different splice variants expressed in the different strains, as well as the susceptibility (S) or resistance (R) to F-MLV infection. Data taken from references [7],[8],[65],[66]. (B) Different *A3* alleles found in human populations. At least seven SNP polymorphisms have been found in the A3G genes in humans, although only one polymorphic *A3G* allele, H186R, has been associated with increased susceptibility to HIV-1-mediated disease [69]. Also shown is the presumed recombination event leading to the deletion of part of A3B in some individuals and a fused h*A3A*/*3B* gene [71].

A3 proteins belong to a family of genes that encode DNA- and RNA-editing enzymes and confer innate immunity to HIV-1 and perhaps other viruses, such as hepatitis B virus (HBV) and human papilloma virus (HPV) [9],[10]. The A3 genes arose through gene duplication of a single-copy primordial gene, are found in a tandem array, and have expanded or contracted in different species [11]; the human genome encodes seven *A3* (*hA3*) genes, the feline four genes [12], the horse six genes [13], artiodactyls species two to three genes [14], and the mouse genome a single *A3* (*mA3*) gene [15]. The *A3* genes in general show a high degree of polymorphic variation, suggesting that they are under strong selective pressure [8], [12], [16]–[19]. Additionally, alternatively spliced RNAs with the potential for generating different A3 proteins have been found in the mouse (Figure 1A; see below), felines [12], and artiodactyls [14].

hA3G, the first identified family member, was discovered because of its interaction with the HIV-1 virion infectivity factor (Vif) [5]. hA3G and subsequently hA3F were shown to inhibit HIV-1 lacking the *vif* gene. In *vif*-deficient HIV-1 producer cells, both hA3G and hA3F are packaged into progeny virions via interaction with the nucleocapsid (NC) protein and viral RNA. Once packaged, hA3 proteins inhibit infection in target cells by deaminating deoxycytidine residues on the DNA minus strand following reverse transcription, inducing G to A hypermutation in newly synthesized HIV-1 DNA. A3 proteins also inhibit replication by cytidine deaminase (CDA)-independent mechanisms [20]. In cells infected with *vif*+ HIV-1, Vif binds hA3G and hA3F and targets these proteins for ubiquitinylation and degradation in the proteosome, thereby overcoming the anti-viral activity [21]–[24]. Simian immunodeficiency viruses (SIVs) also encode Vif proteins, while foamy viruses (FVs) encode a protein (Bet) that interacts with A3G and prevents its packaging via a mechanism that is apparently different from that of Vif [25]–[27]. There are also a large number of studies demonstrating that A3 proteins inhibit transposition of human and mouse retroelements, such as LINE-1, Alu, MusD, human endogenous retroviruses (HERVs), and IAPs [28]–[34]. Although no study has directly examined the effect of A3 proteins on endogenous copies of these elements, both HERV and endogenous murine leukemia virus (MLV) sequences bear signatures of cytidine deamination [35]–[37].

Several studies have examined packaging of A3 proteins from different species into retroviruses endemic to the species using transfected tissue culture cells and have suggested that these viruses are resistant to the A3 proteins of their natural hosts. For example, it has been shown that human T cell leukemia virus I (HTLVI), Mason Pfizer monkey virus (MPMV), and MLV do not efficiently package human, monkey, or mouse A3 proteins, respectively, because of weak interactions between the NC proteins and the host A3 [38]–[46], although other studies have shown some packaging of host A3 proteins by HTLVI and MLV as well as viral restriction [47]–[50]. The MLV protease may also degrade mA3, thereby preventing its anti-viral function [45]. More recently, equine infectious anemia virus was shown to package several horse A3 proteins but these did not diminish infection as effectively as human A3 proteins [13].

In contrast to studies demonstrating that endogenous A3 proteins do not restrict retroviruses that infect the same species, there are many examples of cross-species restriction in cultured cells. Human A3B and A3C restrict SIV [51] and hA3G restricts Rous sarcoma virus, feline FV, MLV, and mouse mammary tumor virus (MMTV) [6], [25], [42]–[44], [48], [52]–[54]. Indeed, several human A3 proteins including hA3G have been shown to restrict MLV via deamination [42],[43],[54],[55], although to a lesser extent than HIV-1 [50]. There is also species-specific degradation of hA3G proteins by different Vifs. For example, Vifs encoded by SIVs that infect humans (chimpanzees and sooty mangabeys) can cause the degradation of hA3G, while Vifs from SIVs that don't infect humans (African green monkeys) don't interact with hA3G [56]–[59]. Additionally, mouse A3, which does not bind Vif, restricts HIV-1 infection [48], MPMV [40], and primate FV [27] via a CDA-dependent mechanism. Thus, it has been proposed that one role for A3 proteins is to prevent zoonoses rather than to restrict natural infections [9].

However, the studies showing that A3 proteins do not restrict viruses that infect the same species need to be re-evaluated in light of several recent studies with two mouse viruses, MMTV and MLV. These in vivo studies examined the role of A3 in the resistance and susceptibility to infection in different inbred strains of mice and in mice with targeted deletion of the m*A3* gene and clearly demonstrate that the host A3 protein plays a role in virus infection and, more importantly, in virus-mediated pathogenesis.

In the first of these studies, our lab tested whether knockout mice that lack a functional A3 gene were susceptible to infection with MMTV [6]. MMTV is a betaretrovirus that is normally acquired through milk by suckling neonates and first infects cells in the lymphoid compartment followed by transmission to the mammary epithelial cells [60]. We infected mA3+/+ and mA3−/− mice and showed that mA3−/− mice had higher levels of initial infection compared to their wild-type littermates and moreover, that virus spread was more rapid and extensive. We have also found that MMTV-infected mA3−/− mice develop mammary tumors more rapidly (C. Okeoma and S. Ross, unpublished data). These findings demonstrate that mA3 provides partial protection to mice against MMTV infection and represent the first demonstration to our knowledge that A3 proteins function during in vivo retroviral infection.

Two groups more recently demonstrated that mA3 also restricts Friend MLV (F-MLV) infection and virus-induced erythroproliferation [7],[8]. In the 1970s, genetic crosses between various strains of inbred mice were carried out as a means of identifying genetic loci that would confer susceptibility or resistance to F-MLV [61]. The recovery from Friend virus 3 (*Rfv*3) locus was subsequently found to affect the ability of resistant mice, such as C57BL/6, to recover from virus infection, at least in part through the production of a high-level antibody response [62],[63]. Mapping studies placed *Rfv*3 to a 0.83 centimorgan region of chromosome 15, close to the *APOBEC3* locus [64],[65]. The Greene and Miyazawa groups tested whether *Rfv*3 and m*A3* were one and the same, and their data suggest that this may be the case [7],[8]. Both groups showed that mA3−/− mice were more highly infected by F-MLV than were mA3+/+ mice, and when F-MLV-susceptible (BALB/c) mice were crossed with mA3−/− mice and infected with virus, the F1 progeny had high virus titers, did not develop a strong neutralizing antibody response, and showed increased erythroid cell proliferation characteristic of F-MLV-mediated disease. Conversely, mA3−/− X C57BL/6 F1 mice looked like their resistant parent, with low viremia and high antibody production.

The two groups differed in their conclusions regarding the mechanism of A3-mediated resistance. There are a number of differences between the A3 alleles in F-MLV-resistant and –susceptible mice, leading to differences in mRNA expression levels, alternative splicing of the mRNA, and amino acid polymorphisms [7],[8],[45],[65]. C57BL/6 mice predominantly express a Δexon 5 variant of mA3 (Figure 1A) [7],[8],[66]. Using a transfection/infection assay with hybrid A3 molecules, Takeda and colleagues mapped the ability of the C57BL6-derived allele to restrict infection to polymorphic amino acids in the N-terminal 192 amino acid region and not to the absence of exon 5. In contrast, Santiago and colleagues suggested that a lack of exon 2 in the mA3 made in BALB/c (or A.BY) susceptible mice contributes to the inability of mA3 from these backgrounds to inhibit infection, although this transcript was not detected in the studies by Takeda et al. and our lab has more recently found that the major transcript in BALB/c mice contains exon 2 [66]. C57BL/6 mice also express higher levels of A3 RNA than BALB/c mice [7],[65],[66]. We also found that the C57BL/6 mA3 allele restricts MMTV infection more effectively than that encoded in BALB/c mice [66]. Which of these differences contribute to resistance to virus infection in vivo awaits the creation of transgenic or knock-in mice with the different alleles to directly test their efficacy in restricting F-MLV and MMTV.

Importantly, these studies demonstrate that the tissue culture experiments that examine the role of host restriction factors can underestimate the role that these restriction factors play in vivo. The numerous studies regarding mA3's effect on MLV infection in cultured cells are conflicting, providing evidence for and against a role in restriction [43],[44],[49],[50],[67]. Although the tissue culture studies predominantly examined the role of mA3 on Moloney MLV (M-MLV) rather than F-MLV, a recent study also shows that mA3−/− mice are more susceptible than mA3+/+ mice to infection and lymphoma induction by M-MLV [68]. While the F-MLV studies do not definitively prove that *Rfv3* is *mA3*, the fact that mA3−/− mice are more susceptible to infection by at least two murine retroviruses, MMTV and MLV, and that the loss of this gene in vivo leads to increased pathogenesis by these viruses, provides strong support that this host-encoded restriction factor does function against a natural pathogen.

What does this mean for HIV-1 and other human pathogens and the role that hA3 proteins play in restricting HIV-1 infection? There is increasing genetic evidence that A3 proteins protect against infection by HIV-1 and other viruses. Indeed, one of the alleles that has been linked to individuals who have received multiple exposures to HIV but remained sero-negative maps to chromosome 22q12-13, which contains the human A3 family member genes [64],[65]. One A3G polymorphism, H16R, is associated with AIDS progression and declining CD4 T cells, although the in vitro anti-viral activity of the two alleles was the same [69]. Several human *A3* genes, particularly *A3G* and *A3H*, are highly polymorphic, suggesting positive selection by viruses or retroelements [18],[19],[70], and there is a known deletion in the *A3B* locus that leads to a fused h*A3A*/*3B* gene [71] (Figure 1B). Although alternative splicing has been shown to generate different A3 proteins in mice, artiodactyls, and felines, thus far only APOBEC3H has been shown to undergo alternative splicing in humans [72]. This represents another potential means of generating A3 proteins that more effectively restrict infection and needs to be more thoroughly examined for the different human genes.

The mouse studies underscore the importance of using in vivo models to understand host restriction factors and their importance in limiting viral pathogenesis. Unfortunately, the lack of a good animal model means we can at present only infer that A3G or other A3 molecules are retained in the genome as anti-HIV-1 (or other viruses) restriction factors in humans and other species. In the absence of being able to test species-specific A3 molecules in vivo against species-endemic viruses, it is critical to look for genetic associations between polymorphic *A3* alleles and resistance to infection to HIV-1, HTLVI, HBV, HPV, and other viruses whose infection may be affected by A3 proteins *in vivo*.
